# Nifuroxazide Pretreatment Protects Against Acute CdCl_2_ Exposure-Induced Toxicity via Modulating the USP21/AIM2 Inflammasome Axis

**DOI:** 10.3390/antiox15091211

**Published:** 2026-09-20

**Authors:** Jing Yu, Feng Xu, Ran Wang, Qiu-Man Li, Xiang Li, Yi-Fang Jiang, Jian-Fei Lu, Chang-Hong Li, Guan-Jun Yang, Jiong Chen

**Affiliations:** 1 State Key Laboratory for Managing Biotic and Chemical Threats to the Quality and Safety of Agro-Products, Ningbo University, Ningbo 315211, China; 2411130055@nbu.edu.cn (J.Y.); 2411130037@nbu.edu.cn (R.W.); 256004118@nbu.edu.cn (Q.-M.L.); 2511130038@nbu.edu.cn (X.L.); 2511130027@nbu.edu.cn (Y.-F.J.); lujianfei@nbu.edu.cn (J.-F.L.); lichanghong@nbu.edu.cn (C.-H.L.); 2 Laboratory of Biochemistry and Molecular Biology, Key Laboratory of Applied Marine Biotechnology of Ministry of Education, School of Marine Sciences, Ningbo University, Ningbo 315211, China; 3 Department of Intensive Care Unit & Central Laboratory, The First Affiliated Hospital of Ningbo University, Ningbo 315010, China; xufengnbu@nbdyyy.com

**Keywords:** cadmium, liver, Nifuroxazide, USP21, AIM2 inflammasome, redox homeostasis, pyroptosis

## Abstract

Cadmium (Cd) is a ubiquitous environmental pollutant with an exceptionally long biological half-life (10–30 years) and high multi-organ toxicity. While chronic cadmium exposure has been extensively studied, the pathological mechanisms and prophylactic strategies for acute cadmium poisoning remain poorly defined. Here, we established an acute cadmium intoxication model in C57BL/6 mice via intraperitoneal injection of CdCl_2_ (5 mg/kg) and assessed pathological and molecular changes 12 h post-exposure. Histopathological examination revealed overt hepatic damage and mild extrahepatic changes in kidney and spleen. Mechanistically, acute cadmium challenge disrupted systemic redox homeostasis, as evidenced by significant reductions in superoxide dismutase (SOD), glutathione peroxidase (GSH-PX), and total antioxidant capacity (T-AOC). Concurrently, cadmium triggered a robust inflammatory response, upregulating pro-inflammatory cytokines (IL-1β, IL-6, TNF-α) and lactate dehydrogenase (LDH) release, and activated the pyroptotic pathway, as shown by elevated cleaved caspase-1 and GSDMD-N levels. To identify potential prophylactic agents, we investigated nifuroxazide (NFX), a multifunctional agent with known anti-cancer, antioxidant, and anti-inflammatory properties. Our results indicate that NFX pretreatment protects against cadmium-induced acute liver injury. Mechanistically, these data support the involvement of the USP21–AIM2 axis in this protection. USP21, a deubiquitinase, stabilizes AIM2 by removing its ubiquitin chains. Cadmium exposure increased USP21-mediated AIM2 deubiquitination, decreased AIM2 ubiquitination, and promoted AIM2 protein accumulation, accompanied by AIM2 inflammasome activation and hepatic stellate cell pyroptosis. In contrast, NFX pretreatment was associated with reduced USP21-mediated AIM2 deubiquitination, increased AIM2 ubiquitination and reduced AIM2 protein accumulation, and suppressed hepatic stellate cell pyroptosis. Collectively, these findings indicate that NFX pretreatment protects against acute CdCl_2_-induced hepatic injury with mild improvements in extrahepatic tissue histology, and support the involvement of the USP21–AIM2 axis in regulating AIM2 inflammasome protein stability.

## 1. Introduction

According to authoritative risk-assessment summaries from the Agency for Toxic Substances and Disease Registry (ATSDR) [1] and European Food Safety Authority (EFSA) [2], cadmium (Cd) is a highly bioaccumulative heavy metal with long biological half-life in humans; liver and kidney represent major target organs, where chronic low-dose exposure mainly causes renal tubular injury, whereas high-dose acute exposure can trigger overt hepatic damage [3,4,5,6]. While renal toxicity is the predominant long-term consequence of chronic cadmium exposure, acute cadmium-induced hepatic injury represents a distinct and pathologically significant scenario that warrants in-depth mechanistic exploration. However, compared with the extensively studied chronic cadmium toxicity, the molecular pathophysiology of acute cadmium-induced liver damage remains less well characterized. As the central organ for systemic metabolism and detoxification, the liver bears the primary burden of processing circulating cadmium and is thus particularly vulnerable to acute toxic insult. Accumulating experimental evidence confirms that acute cadmium exposure induces severe hepatopathology, including vacuolization, nuclear condensation, and necrosis in murine models [7,8,9]. Hence, deciphering the pathophysiological mechanisms of acute liver injury elicited by cadmium and exploring potential protective strategies against cadmium-provoked hepatic damage must now be regarded as an urgent research imperative.

A widely acknowledged early initiator of acute cadmium hepatotoxicity is oxidative stress [4,10,11]. Upon entry, Cd^2+^ ions deplete glutathione, inhibit antioxidant enzymes (e.g., SOD, GSH-PX), and directly impair mitochondrial function, driving excessive reactive oxygen species (ROS) accumulation. These ROS not only trigger lipid peroxidation and organelle damage but also act as upstream signals that aberrantly activate pattern-recognition receptors. Among the downstream effectors, inflammasome assembly and hyperactivation serve as critical links between initial oxidative insult and terminal inflammatory injury [12,13,14]. Recent attention has shifted from the canonical NLRP3 inflammasome to other family members, notably the absent in melanoma 2 (AIM2) inflammasome [15,16,17,18]. Upon sensing cytosolic double-stranded DNA—whether from damaged mitochondria or exogenous sources—AIM2 recruits and activates caspase-1, which cleaves gasdermin D (GSDMD) to form membrane pores, releasing large amounts of IL-1β and IL-18 and driving pyroptosis, a highly pro-inflammatory form of regulated cell death [19,20,21]. However, the precise dysregulation of the AIM2 inflammasome in CdCl_2_-induced acute liver injury, and the contribution of AIM2-mediated pyroptosis to hepatic pathological damage, remain poorly understood.

Deubiquitinating enzymes (DUBs) finely tune inflammatory signaling by removing ubiquitin chains from substrates, thereby modulating protein stability, activity, and localization [22,23]. Ubiquitin-specific protease 21 (USP21), a member of this family, has been implicated in innate immune regulation in specific cell types. Notably, in resting human macrophages, AIM2 is constitutively ubiquitinated and targeted for proteasomal degradation to prevent spontaneous inflammation; USP21-mediated deubiquitination is essential for AIM2 inflammasome assembly [24]. Nevertheless, whether USP21 similarly regulates AIM2 in hepatic stellate cells, and its role in cadmium hepatotoxicity, has yet to be investigated.

Nifuroxazide (NFX), a safe nitrofuran antimicrobial agent clinically indicated for acute diarrheal diseases [25], exhibits minimal systemic absorption and favorable safety profiles for gastrointestinal infectious conditions [26], has recently garnered attention for its pleiotropic pharmacological activities, including anticancer, antioxidant, and anti-inflammatory effects. In non-malignant inflammatory models, NFX ameliorates LPS-induced myocardial and lung injury by reducing inflammatory burden, lowering serum damage markers (LDH, ALP), and improving oxidative status [27]. In cholestatic liver dysfunction, NFX attenuates inflammation, restores bile acid transport, improves redox balance, and reduces hepatocellular proliferation via inhibition of the IL-6/β-catenin pathway [28]. Despite these promising findings, the role of NFX in acute heavy metal–induced hepatotoxicity remains unexplored.

In this study, we employed a CdCl_2_-induced acute liver injury model in mice to descriptively characterize the hepatic effects of cadmium exposure, including the influence of NFX pretreatment and associated molecular changes, with particular attention to the USP21–AIM2–pyroptosis axis. For in vitro documentation of NFX-related changes in this axis, we used the human hepatic stellate cell line LX-2, a well-characterized hepatic inflammatory effector cell line that expresses multiple core inflammasome components, including AIM2 [29,30,31]; this model was selected for its relevance to hepatic inflammatory signaling rather than for hepatocyte-specific toxicity. This descriptive design was not intended to test predefined hypotheses or to dissect specific toxicological mechanisms; rather, it aimed to comprehensively document cadmium-related effects and thereby provide an informational foundation for future mechanistic and therapeutic investigations.

## 2. Materials and Methods

### 2.1. Establishment of the Experimental Model

**Figure 1:**
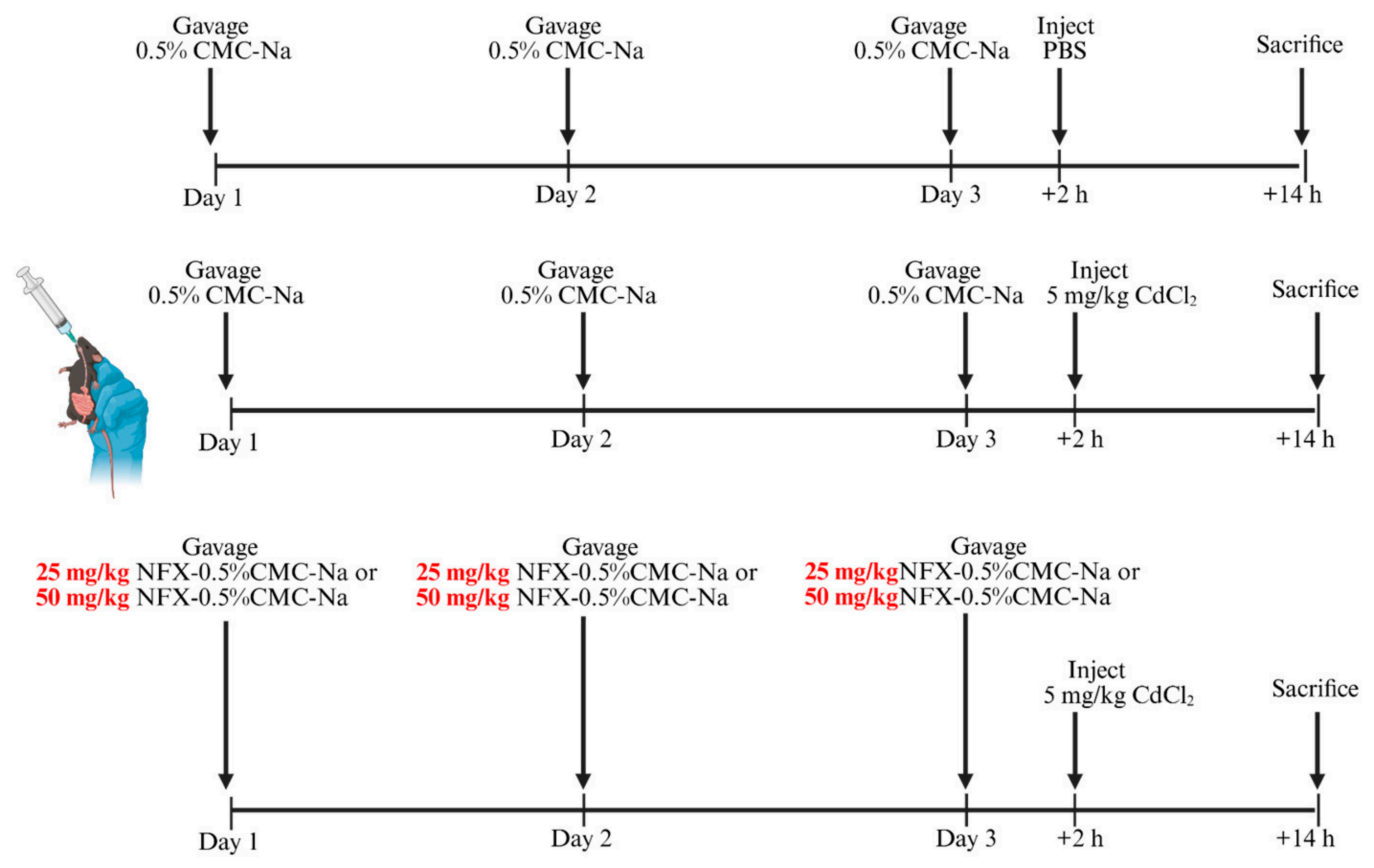
Establishment of an acute cadmium poisoning model. The experimental mice were divided into four groups: the control group, the CdCl_2_ group, the CdCl_2_ + NFX-L group, and the CdCl_2_ + NFX-H group. NFX was dissolved in 0.5% CMC-Na solution and administered via continuous oral gavage for 3 days. Two hours after the third day’s gavage, CdCl_2_ was administered via intraperitoneal injection; 12 h after the injection, whole blood, liver, kidney and spleen samples were collected from the mice. Created in BioRender. Yang, G. (2026) https://BioRender.com/1nczwrb, accessed on 28 July 2026.

Male SPF C57BL/6 mice (4–6 weeks, 18–22 g) from Shanghai Slake Laboratory Animal Co., Ltd. (Shanghai, China) were housed at 22–26 °C, 40–60% humidity, with a 12 h light/dark cycle and free access to sterile food and water. After one week of acclimatization, they were randomly divided into four groups (*n* = 6 in each group), with the allocation process based on a computer-generated sequence of random numbers: control, CdCl_2_ (5 mg/kg, i.p.), CdCl_2_ + low-dose NFX (25 mg/kg, gavage), and CdCl_2_ + high-dose NFX (50 mg/kg, gavage). NFX was suspended in 0.5% CMC-Na and administered daily by gavage for 3 days. On day 3, 2 h after the last gavage, mice (except controls) received a single i.p. injection of CdCl_2_ (5 mg/kg) and were euthanized 12 h later (Figure 1). Liver, spleen, kidney, and intestinal contents were collected; portions of the tissues were fixed for paraffin sectioning, and the remaining tissues and intestinal contents were snap-frozen in liquid nitrogen and stored at −80 °C. Due to the nature of the intervention, personnel who performed the animal treatments were aware of group allocation. These personnel were not involved in outcome assessment, histological evaluation, image quantification, or statistical analysis. All procedures followed China’s regulations for laboratory animal management and were approved by the Animal Ethics Committee of Ningbo University on 24 June 2025 (Approval No. 15603).

### 2.2. Cell Culture

Human hepatic stellate cells (LX-2) were cultured in LX-2-specific medium (Haixing Biotechnology, Hangzhou, China) at 37 °C in a humidified atmosphere containing 5% CO_2_. Cells were divided into five groups: control, CdCl_2_ (20 μM), CdCl_2_ + NFX-L (1 μM), CdCl_2_ + NFX-H (3 μM), and CdCl_2_ + Dex (1 μM; positive control).

### 2.3. Liver Function Tests

In vivo hepatic injury was evaluated by measuring alanine aminotransferase (ALT) and aspartate aminotransferase (AST) levels in mouse serum using commercial assay kits (Abbkine Scientific Co., Ltd., Wuhan, China) according to the manufacturer’s instructions.

### 2.4. Antioxidant Enzyme Activity

For biochemical assessment, serum was prepared, and the levels of total antioxidant capacity (T-AOC), superoxide dismutase (SOD), glutathione peroxidase (GSH-PX), and malondialdehyde (MDA) were quantified using commercial assay kits (Abbkine Scientific Co., Ltd., Wuhan, China) according to the manufacturer’s instructions.

### 2.5. Enzyme-Linked Immunosorbent Assay

Following the manufacturer’s protocols, serum concentrations of IL-1β, IL-18, and leukocyte-derived chemokine 2 (LECT2) were quantified using a commercially available ELISA kit (Wuhan Fine Biotech Co., Ltd., Wuhan, China). This method enables a comprehensive assessment of the systemic inflammatory response in a CdCl_2_-induced acute liver injury model.

### 2.6. Histopathological Assessment

Liver, kidney, and spleen tissues were fixed in 10% neutral buffered formalin for at least 24 h, then processed through routine dehydration, embedded in paraffin, and cut into 4 μm thick sections. Three mice per group were randomly selected for histological analysis. Two non-overlapping fields per mouse were imaged and quantified. Following H&E staining of consecutive sections for routine morphological evaluation, hepatic lesions—specifically pericentral lobular inflammation and centrilobular necrosis—were semiquantitatively graded on a 0-to-3 scale (0 = absent, 1 = mild, 2 = moderate, 3 = marked) [32,33]. The pathological scoring of kidney and spleen tissues was based on the methods described by Manautou et al. [34] and Gao et al. [35], respectively. Slides were coded, and histopathological assessment was performed by two independent observers blinded to group allocation. Briefly, three sections were randomly selected per group, and two microscopic fields per section were examined. Pathological changes in each organ were then scored and graded according to their severity.

### 2.7. TUNEL Assay

To detect TUNEL-positive cell death, paraffin-embedded liver sections were subjected to terminal deoxynucleotidyl transferase dUTP nick-end labeling (TUNEL) using a commercial kit (Beyotime Biotechnology, Shanghai, China). Three mice per group were randomly selected for histological analysis. Two non-overlapping fields per mouse were imaged and quantified. Nuclei were counterstained with DAPI (for fluorescence) or hematoxylin (for chromogenic detection), and images were acquired using a light or fluorescence microscope as appropriate. TUNEL-positive cells were identified by brown (chromogenic) or green (fluorescent) nuclear staining, and apoptosis was quantified by calculating the percentage of TUNEL-positive hepatocytes relative to total hepatocytes in at least five randomly selected high-power fields per section.

### 2.8. Quantitative Real-Time PCR (RT-qPCR)

Total RNA was extracted from ~0.1 g mouse liver tissue with TRIzol^®^ reagent (Invitrogen, Carlsbad, CA, USA) using chloroform phase separation, followed by isopropanol precipitation, a 75% ethanol wash, air-drying, and dissolution in DEPC-treated water. RNA concentration and purity were checked by NanoDrop spectrophotometer (Thermo Fisher Scientific, Wilmington, DE, USA; A260/A280 ratio 1.8–2.0). First-strand cDNA was synthesized from 1 μg total RNA using the PrimeScript^TM^ RT Kit (RR037A, TaKaRa, Beijing, China) in a 20 μL reaction according to the manufacturer’s protocol.

RT-qPCR was performed on a QuantStudio 3 system (Thermo Fisher Scientific, Waltham, MA, USA) using BeyoFast SYBR Green Master Mix (Beyotime, Shanghai, China) in 20 μL reactions. Cycling conditions: 95 °C for 2 min; 40 cycles of 95 °C for 15 s and 60 °C for 30 s; followed by a dissociation stage. β-actin served as the reference gene, and relative mRNA levels were calculated by the 2*^−^*^ΔΔ*Ct*^ method. All reactions were run in triplicate; primer sequences are listed in Table S1. Three biologically independent samples per group were used. Each sample was run in triplicate (technical replicates), and the triplicate values were averaged before statistical analysis.

### 2.9. Hoechst33342/PI Double Staining Assay

Following seeding into 6-well plates at 2 × 10^5^ cells per well, LX-2 cells were pretreated with NFX (1 or 3 μM) or dexamethasone (1 μM) for 2 h, then challenged with 20 μM CdCl_2_ for 24 h. Cell death was assessed by Hoechst 33342 and propidium iodide (PI) double staining based on Ansari [36,37]. Specifically, adherent cells were rinsed with PBS, incubated with Hoechst 33342 (MedChemExpress, Monmouth Junction, NJ, USA; 10 μg/mL, 15 min, room temperature) and subsequently with PI (20 μg/mL, 15 min) in the dark. Three PBS washes preceded fixation in 4% paraformaldehyde for 30 min, after which cells were observed under a fluorescence microscope. Image acquisition and merging employed ImageJ software (version 1.53k; NIH, Bethesda, MD, USA), and the percentage of PI-positive cells was quantified as an indicator of loss of membrane integrity/cell death.

### 2.10. CETSA-Based Target Screening

Following harvest and two washes with ice-cold PBS, LX-2 cells (1 × 10^7^) were lysed on ice for 30 min in 600 μL of ice-cold lysis buffer (50 mM Tris-HCl pH 7.5, 150 mM NaCl, 1% NP-40, 1% protease inhibitor cocktail, and 1% phosphatase inhibitors). Insoluble debris was removed by centrifugation (12,000× *g*, 10 min, 4 °C), and the resulting supernatant was divided into two equal aliquots. One aliquot was treated with 3 μM NFX (dissolved in DMSO; final DMSO < 0.1%), while the other received an equal volume of DMSO as vehicle control; both were incubated on ice for 30 min. Each aliquot was then subdivided into two equal portions and heated for 8 min at 60 and 65 °C using a thermocycler (Bio-Rad Laboratories, Hercules, CA, USA). After heating, samples were immediately cooled on ice for 5 min and centrifuged (12,000× *g*, 10 min, 4 °C) to pellet denatured/aggregated proteins. The soluble supernatants were collected, mixed with 5× SDS loading buffer, boiled for 5 min, and resolved by SDS-PAGE on 10–12% polyacrylamide gels, followed by silver staining [38,39,40].

### 2.11. CETSA-Based Target Validation

Based on the above screening results and guidance from molecular docking predictions, the candidate targets were further validated using a combination of gradient CETSA and Western blot analysis. Lysates from the drug-treated and control groups were each dispensed into multiple PCR tubes, and a temperature gradient of 55–80 °C (e.g., 55 °C, 60 °C, 65 °C, 70 °C, 75 °C, 80 °C) was set, with each temperature maintained for exactly 7 min. Subsequently, the samples were centrifuged at 4 °C and 20,000× *g* for 20 min, and the supernatant was collected for Western blot analysis to assess the levels of the putative target proteins. Compared with the control group, the melting curves (*T_m_*) of the NFX-treated samples shifted to the right, which was interpreted as a sign of direct or indirect target binding [41,42].

### 2.12. Kyoto Encyclopaedia of Genes and Genomes and Gene Ontology Enrichment Analysis

Publicly available microarray dataset GSE302882 from GEO (https://www.ncbi.nlm.nih.gov/geo/query/acc.cgi?acc=GSE302882; accessed on 26 May 2026) was re-analyzed to identify genes associated with CdCl_2_-induced acute liver injury. Following normalization, differentially expressed genes (DEGs) were identified via GEO2R and/or limma (|log_2_ fold change| > 1, adjusted *p* < 0.05). GO and KEGG enrichment analyses were performed using DAVID (https://davidbioinformatics.nih.gov/, accessed on 26 May 2026) and clusterProfiler (*p* < 0.05, FDR < 0.05). A PPI network was built with STRING (https://string-db.org/; accessed on 26 May 2026), followed by functional re-evaluation of hub genes. Pathways consistently enriched across analyses—especially those linked to inflammation, oxidative stress, and metabolic disorders—were prioritized for subsequent experimental validation.

### 2.13. Molecular Docking

Ligand 2D structures from PubChem were converted to 3D models with ChemOffice 2.0, and protein crystal structures were retrieved from the RCSB PDB. After removal of water molecules and phosphate groups in PyMOL 2.6.0, energy minimization was performed using MOE 2019. Molecular docking (50 independent runs) was then conducted in MOE 2019, with binding affinities ranked by energy scores. Docking results were visualized with PyMOL 2.6.0 and Discovery Studio 2019 [39].

### 2.14. Western Blotting

Upon lysis of cells in RIPA buffer containing protease inhibitors, protein concentrations were measured using the BCA assay. Equal protein aliquots were resolved on 15% SDS-PAGE gels, electrotransferred to PVDF membranes, and subjected to blocking with 5% skimmed milk. Subsequent incubation with primary antibodies (Table S2) was followed by detection with HRP-conjugated secondary antibodies. Protein bands were visualized using enhanced chemiluminescence (ECL) substrate, and band intensities were quantified using ImageJ software. Three biologically independent samples per group were used. Each sample was run in triplicate (technical replicates), and the triplicate values were averaged before statistical analysis.

### 2.15. Co-Immunoprecipitation and Ubiquitination Assay

Seeded at 3 × 10^6^ cells per 6 cm dish, LX-2 cells were cultured for 24 h in their specific medium and grouped according to Method 2.2. After 24 h of drug treatment, cells received a 4 h pretreatment with the proteasome inhibitor MG132 (10 μM) and the deubiquitinating enzyme inhibitor PR-619 (10 μM) before collection. Cells were lysed in RIPA buffer supplemented with protease inhibitors and N-ethylmaleimide (NEM) to preserve protein ubiquitination. Lysates were centrifuged at 12,000× *g* for 15 min at 4 °C, and the supernatant was collected. Total protein concentration of each lysate was quantified using the BCA assay. For immunoprecipitation, equal total protein amounts of cell lysate from each group were separately incubated with anti-AIM2 antibody or anti-USP21 antibody for 4 h at 4 °C. Protein A/G magnetic beads were then added, and samples were rotated overnight at 4 °C. The captured immune complexes were washed thoroughly, followed by heat elution at 70 °C. Equal volumes of immunoprecipitated eluates were loaded for Western blotting. For anti-AIM2 immunoprecipitation, ubiquitin signals within AIM2 immunoprecipitates were detected. For anti-USP21 immunoprecipitation, anti-AIM2 and anti-ubiquitin antibodies were used to detect AIM2 protein and ubiquitin signals present in USP21 immunoprecipitates, respectively.

### 2.16. USP21 siRNA Transfection

LX-2 cells were seeded and cultured in six-well plates, and cell transfection was performed when the cell confluence reached approximately 70%. The small interfering RNA targeting USP21 (si-USP21) and commercial universal negative control siRNA (si-NC) were chemically synthesized by GenePharma (Shanghai, China). The detailed sequences of si-USP21 [43] are listed in Table 1, while the sequence of si-NC is proprietary and not publicly available. siRNA transfection was conducted using Lipo8000^TM^ transfection reagent (Beyotime Biotechnology, Shanghai, China) in strict accordance with the manufacturer’s standard operating procedures.

### 2.17. USP21 Knockdown Experiment

To investigate whether USP21 mediates the protective effects of NFX against Cd-induced hepatic stellate cell injury, we performed USP21 knockdown via siRNA transfection. LX-2 cells were seeded in six-well plates and transfected with si-USP21 or negative-control siRNA (si-NC) at ~70% confluence as described in Section 2.15. At 22 h post-transfection, each siRNA-transfected cell pool was further divided into three treatment subgroups: untreated blank control, CdCl_2_-exposed model (20 μM CdCl_2_), and NFX-intervention (3 μM NFX 2 h pretreatment prior to CdCl_2_ stimulation). After CdCl_2_ stimulation for 24 h (48 h total post-transfection), cells were harvested for total protein and total RNA isolation. Changes in AIM2 abundance, AIM2 ubiquitination level and cellular pyroptosis markers were assessed.

For statistical evaluation of whether the NFX effect depends on USP21, a 2 × 2 factorial dataset derived only from Cd-challenged subgroups was analysed by two-way ANOVA, with two factors: siRNA status (si-NC/si-USP21) and NFX treatment (-NFX/ + NFX). Special attention was paid to the siRNA-by-NFX interaction term. The blank control subgroups were only included for baseline phenotypic reference and were not incorporated into this two-way ANOVA interaction analysis. This setup allows us to evaluate to what extent USP21 knockdown modulates the protective responses conferred by NFX upon cadmium insult.

### 2.18. Statistical Analysis

Statistical analyses were performed on coded data, and the statistician was blinded to group allocation until the final analysis was completed. Data are presented as mean ± SD. Statistical analyses were performed using GraphPad Prism 9.5 (GraphPad Software, San Diego, CA, USA). Before parametric testing, data distribution was assessed using the Shapiro–Wilk test for normality and Levene’s test for homogeneity of variance. For comparisons between two groups, Student’s t-test was used when assumptions were met; otherwise, the Mann–Whitney U test was applied. For comparisons among three or more groups, one-way ANOVA followed by Tukey’s post hoc test was used when data were normally distributed with equal variances. When two factors were involved, two-way ANOVA with Tukey’s post hoc test was used. For the USP21 knockdown experiment specifically, an additional two-way ANOVA was performed exclusively on the four CdCl_2_-exposed subgroups to test the siRNA-by-NFX interaction term, with untreated blank control groups excluded from this interaction analysis. Full two-way ANOVA outputs (main effects and interaction *p*-values) are summarized in Table S3. For non-parametric data, the Kruskal–Wallis test followed by Dunn’s multiple comparison test was applied. For the reanalysis of public transcriptomic data (GSE302882), differentially expressed genes were identified using the limma package, and *p* values were adjusted for multiple testing using the Benjamini–Hochberg method. A two-sided *p* value < 0.05 was considered statistically significant. Significance symbols are defined as follows: ^#^ *p* < 0.05, ^##^ *p* < 0.01, ^###^ *p* < 0.001, ^####^ *p* < 0.0001 vs. CdCl_2_ group; * *p* < 0.05, ** *p* < 0.01, *** *p* < 0.001, **** *p* < 0.0001 vs. control group. These figure symbols correspond to post hoc pairwise comparisons from one-way ANOVA; main effect and interaction *p*-values from the two-way ANOVA interaction analysis are reported in the main text and Table S3.

## 3. Results

### 3.1. NFX Alleviates CdCl_2_-Induced Hepatic Histopathological Damage and Hepatocyte Death, with Mild Protective Effects in Extrahepatic Organs

**Figure 2:**
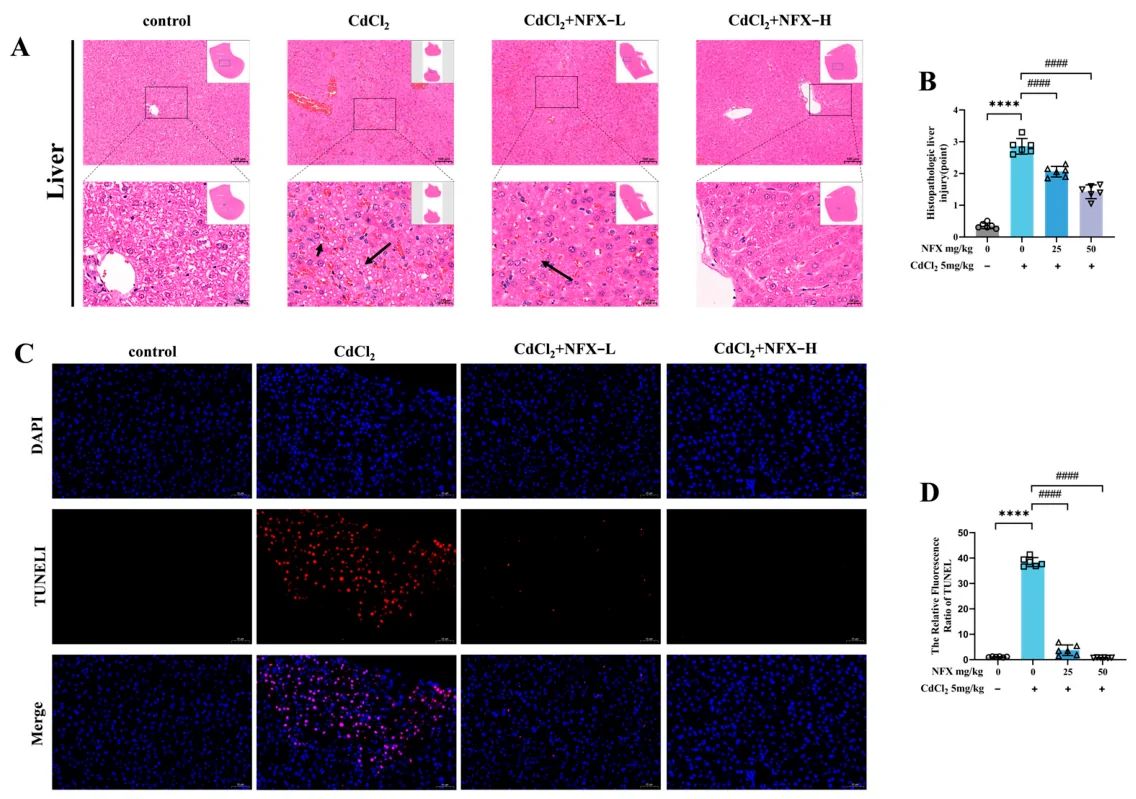
Histopathology of mouse liver, and TUNEL staining of the liver. (**A**,**B**) Histopathological sections of mice liver under different treatment conditions (H&E staining). The top image in each set shows the field of view under low magnification (10×), whilst the bottom image shows a high-magnification view (40×) of the boxed area. (**B**) Histopathological scoring of H&E-stained liver sections. (**C**) TUNEL staining of mice liver tissue (20×). (**D**) Quantification of TUNEL-positive cells in liver. Liver section: → indicates areas of fatty degeneration. The six data points shown in the figure represent 3 animals × 2 fields per animal; statistical analysis was based on the average of the two fields from each animal, with *n* = 3 animals per group. ^####^ *p* < 0.0001 vs. CdCl_2_ group; **** *p* < 0.0001 vs. control group.

The protective effects of NFX pretreatment against CdCl_2_-induced tissue injury were evaluated by histopathological examination of the liver following H&E staining (Figure 2A). In the Control group, liver sections exhibited clear hepatic lobular architecture, regularly arranged hepatic cords, and normal hepatocyte morphology. In contrast, CdCl_2_-exposed mice showed marked hepatic cord atrophy, cytoplasmic vacuolation (black arrows), and fatty degeneration. Low-dose NFX pretreatment reduced hepatic cord atrophy and steatosis, resulting in a more regular hepatocyte arrangement. High-dose NFX pretreatment largely restored normal hepatic architecture and attenuated steatosis.

In the spleen and kidney, CdCl_2_ exposure induced only mild histological alterations consistent with systemic toxicity, and these mild changes were partially attenuated by NFX pretreatment; detailed results are provided in Figure S1. Collectively, these findings indicate that NFX pretreatment exerts a robust protective effect against CdCl_2_-induced acute hepatic injury, accompanied by mild ameliorative effects on extrahepatic histological changes.

To further assess hepatocyte death, TUNEL staining was performed on liver sections (Figure 2C). The Control group showed virtually no TUNEL-positive (red fluorescent) cells, indicating minimal baseline apoptosis. The CdCl_2_ model group exhibited abundant red fluorescence and a significant increase in TUNEL-positive cells, indicating marked induction of hepatocyte death. NFX intervention dose-dependently reduced TUNEL-positive cells: the low-dose group showed significantly fewer positive cells than the model group, while the high-dose group exhibited only rare positive cells, with death levels nearly restored to control levels. Assessment of relative TUNEL fluorescence intensity (Figure 2D) revealed a marked increase in the CdCl_2_ group compared with controls. Administration of NFX at both 25 and 50 mg/kg attenuated this signal in a dose-dependent fashion, with the higher dose producing a highly significant reduction relative to the model group. These results indicate that NFX effectively inhibits CdCl_2_-induced hepatocyte death, likely representing a key mechanism underlying its alleviation of hepatic pathological damage

### 3.2. NFX Ameliorates CdCl_2_-Induced Hepatic Dysfunction and Oxidative Stress in Mice

**Figure 3:**
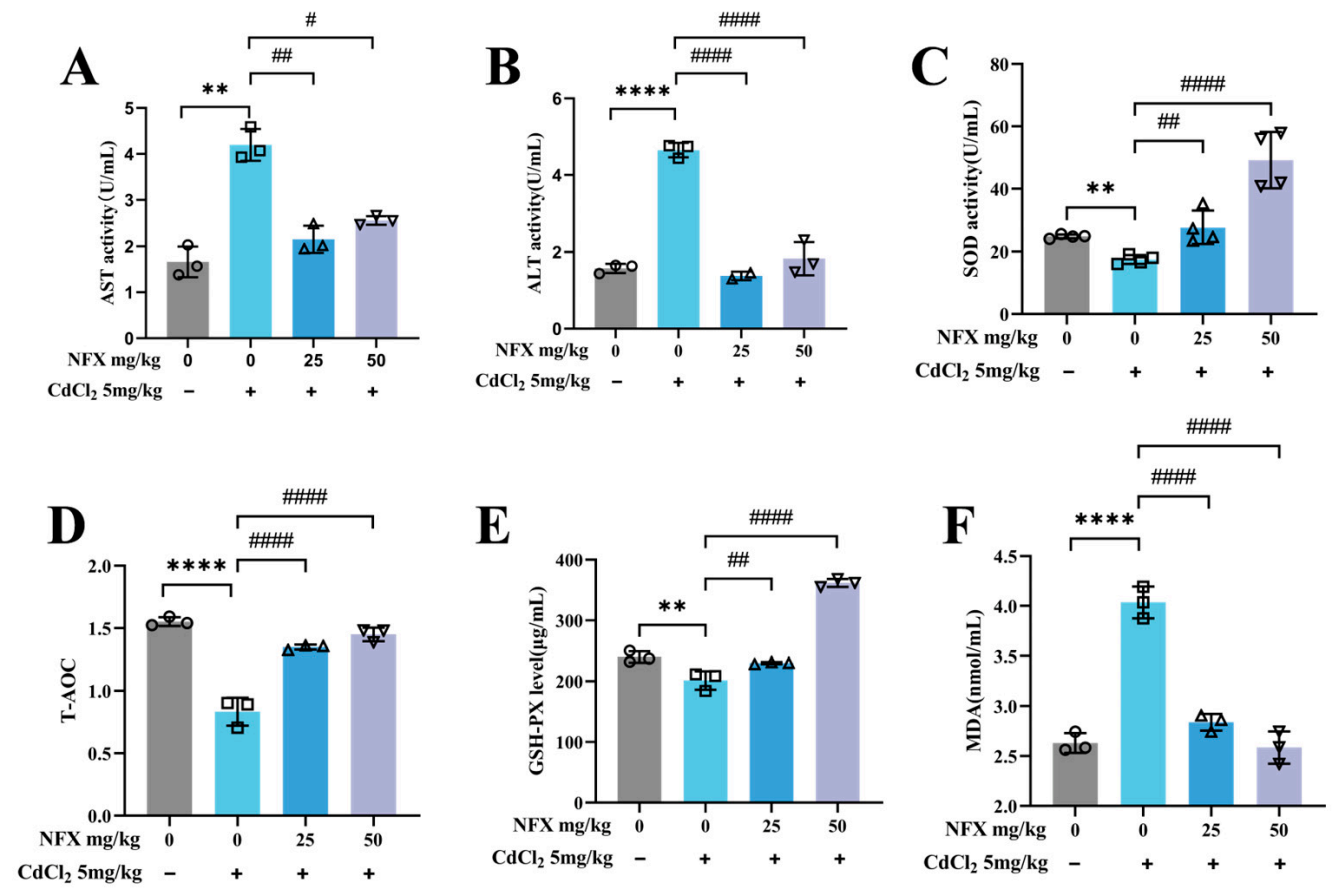
Assessment of lipid metabolism, liver function and antioxidant capacity in mice. (**A**,**B**) ALT and AST levels in the serum of mice in each group; (**C**–**F**) SOD, GSH-PX, T-AOC, MDA levels in the serum of mice in each group. *n* = 3 mice per group. Statistical analysis was performed using one-way ANOVA followed by Tukey’s post hoc test. ^#^ *p* < 0.05, ^##^ *p* < 0.01, ^####^ *p* < 0.0001 vs. CdCl_2_ group; ** *p* < 0.01, **** *p* < 0.0001 vs. control group.

To further evaluate the protective effects of NFX against CdCl_2_-induced liver injury and metabolic disturbances, we measured serum markers of liver function, lipid profile, and oxidative stress (Figure 3). Liver function tests (Figure 3A,B) revealed that serum AST and ALT activities were significantly elevated in the CdCl_2_ model group compared with the Control group, indicating CdCl_2_-induced hepatocyte damage and hepatic dysfunction. NFX treatment dose-dependently reduced AST and ALT activities, with the most significant improvement observed in the CdCl_2_ + NFX-H group, confirming that NFX effectively alleviates CdCl_2_-induced hepatocyte injury.

Oxidative stress assessment revealed that serum SOD activity, GSH-PX levels, and T-AOC were significantly reduced, whereas MDA levels were significantly increased in the CdCl_2_ model group, indicating compromised antioxidant capacity (Figure 3C–F). NFX treatment dose-dependently restored SOD activity and GSH-PX levels, with the high-dose group showing significant improvements compared with the model group, suggesting that NFX effectively counteracts CdCl_2_-induced oxidative imbalance.

Collectively, these findings demonstrate that NFX significantly ameliorates CdCl_2_-induced hepatic dysfunction and oxidative stress imbalance, providing functional support for its subsequent inhibitory effects on inflammatory and pyroptotic pathways.

### 3.3. NFX Suppresses CdCl_2_-Induced Inflammatory Responses and Pyroptosis Pathway Activation in Mouse Livers

**Figure 4:**
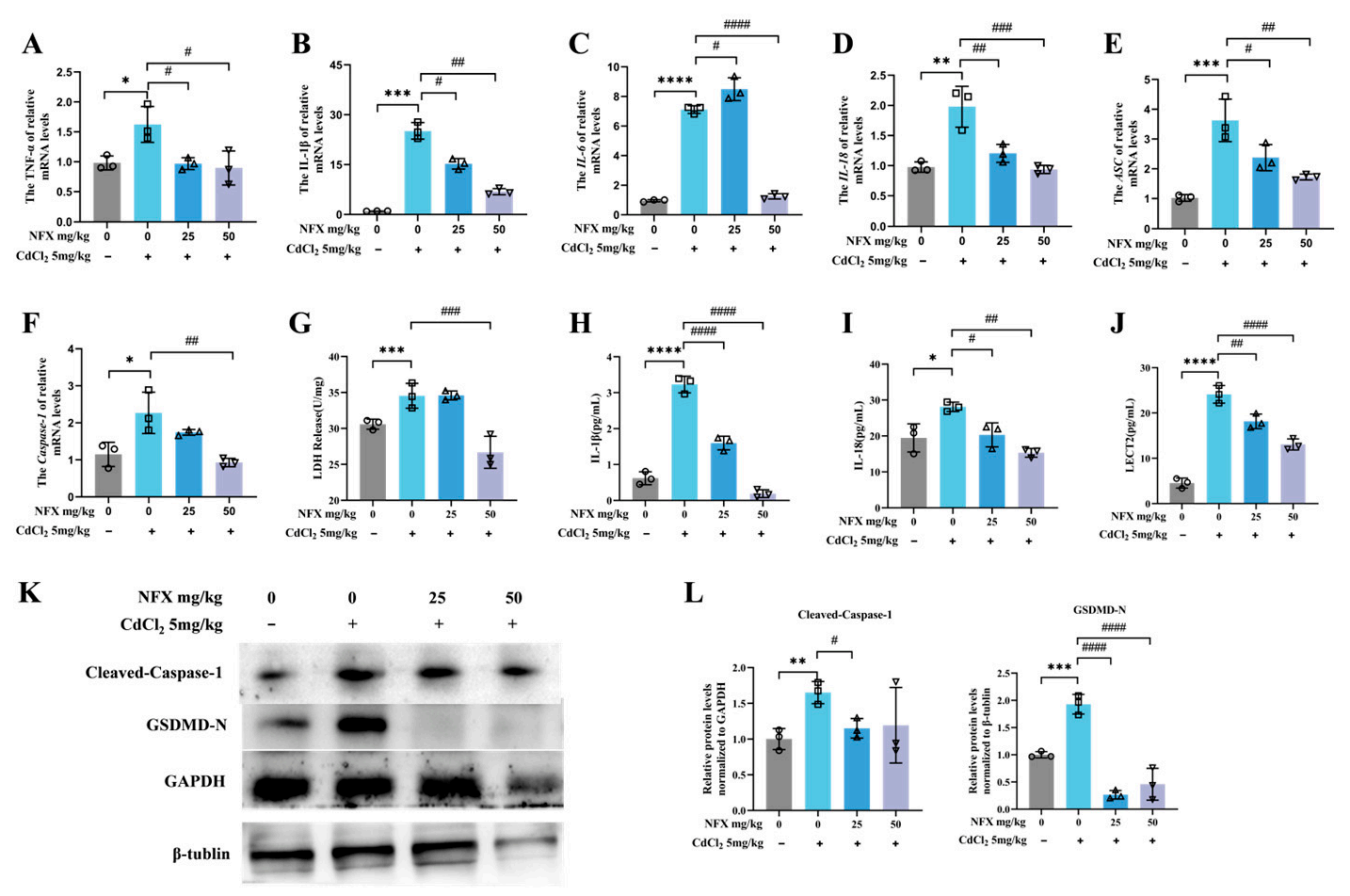
NFX alleviates CdCl_2_-induced acute liver injury in mice by reducing pyroptosis. (**A**–**F**) qRT-PCR quantification of TNF-α, IL-1β, IL-6, IL-18, ASC, and Caspase-1 mRNA levels in mouse liver tissues. (**G**) Measurement of LDH release from mouse liver tissue homogenates. (**H**–**J**) ELISA detection of serum IL-1β, IL-18 and LECT2 levels in mice. (**K**,**L**) Western blot analysis and densitometric quantification of cleaved-caspase-1 and GSDMD-N protein levels in mouse liver tissues. Mice were intragastrically administered with NFX (25 mg/kg or 50 mg/kg) dissolved in 0.5% CMC-Na for three consecutive days. Two hours after the last NFX treatment, mice were intraperitoneally injected with 5 mg/kg CdCl_2_ diluted in normal saline. Liver tissues were collected 12 h after CdCl_2_ injection, immediately snap-frozen in liquid nitrogen and stored at −80 °C. Tissue homogenates were prepared, centrifuged to obtain supernatants, mixed with loading buffer, boiled, and subsequently subjected to immunoblotting. *n* = 3 mice per group. Statistical analysis was performed using one-way ANOVA followed by Tukey’s post hoc test. ^#^ *p* < 0.05, ^##^ *p* < 0.01, ^###^ *p* < 0.001, ^####^ *p* < 0.0001 vs. CdCl_2_ group; * *p* < 0.05, ** *p* < 0.01, *** *p* < 0.001, **** *p* < 0.0001 vs. control group.

To further investigate the regulatory effects of NFX on CdCl_2_-induced hepatic inflammation and pyroptosis, we measured inflammatory cytokine mRNA expression by RT-qPCR, protein secretion by ELISA, and pyroptosis-related protein levels (cleaved caspase-1 and GSDMD-N) by Western blotting (Figure 4). A dose-dependent attenuation of the CdCl_2_-elicited upregulation of inflammatory cytokine mRNAs was observed following NFX administration. Transcript levels of IL-1β, IL-6, IL-18, and TNF-α were markedly lower than those in the model group, with the most substantial reductions occurring in the high-dose cohort. (Figure 4A–D). Similarly, mRNA expression of ASC and Caspase-1 was significantly elevated in the CdCl_2_ model group, whereas NFX intervention effectively downregulated their transcription (Figure 4E,F); Of note, caspase-1 transcript levels in the high-dose NFX group were markedly lower than those measured in the CdCl_2_ group, pointing to an attenuation of pyroptosis-associated gene activation. Immunoblotting results additionally showed that CdCl_2_ challenge strongly upregulated cleaved caspase-1 and GSDMD-N protein abundance, while NFX suppressed these increases in a dose-dependent manner (Figure 4K,L). Additionally, LDH release was significantly elevated in the CdCl_2_ model group (Figure 4G), and serum IL-1β, IL-18 and LECT2 protein levels were markedly increased (Figure 4H–J); NFX treatment dose-dependently reduced LDH release and IL-1β/IL-18 levels, and the high-dose group exhibited marked amelioration relative to the model group. Collectively, these results demonstrate that NFX effectively suppresses CdCl_2_-induced hepatic inflammation and pyroptosis pathway activation, downregulates pro-inflammatory cytokines and key pyroptotic effectors, and alleviates CdCl_2_-induced liver injury.

### 3.4. NFX Attenuates CdCl_2_-Induced Hepatic Stellate Cell Pyroptosis In Vitro

**Figure 5:**
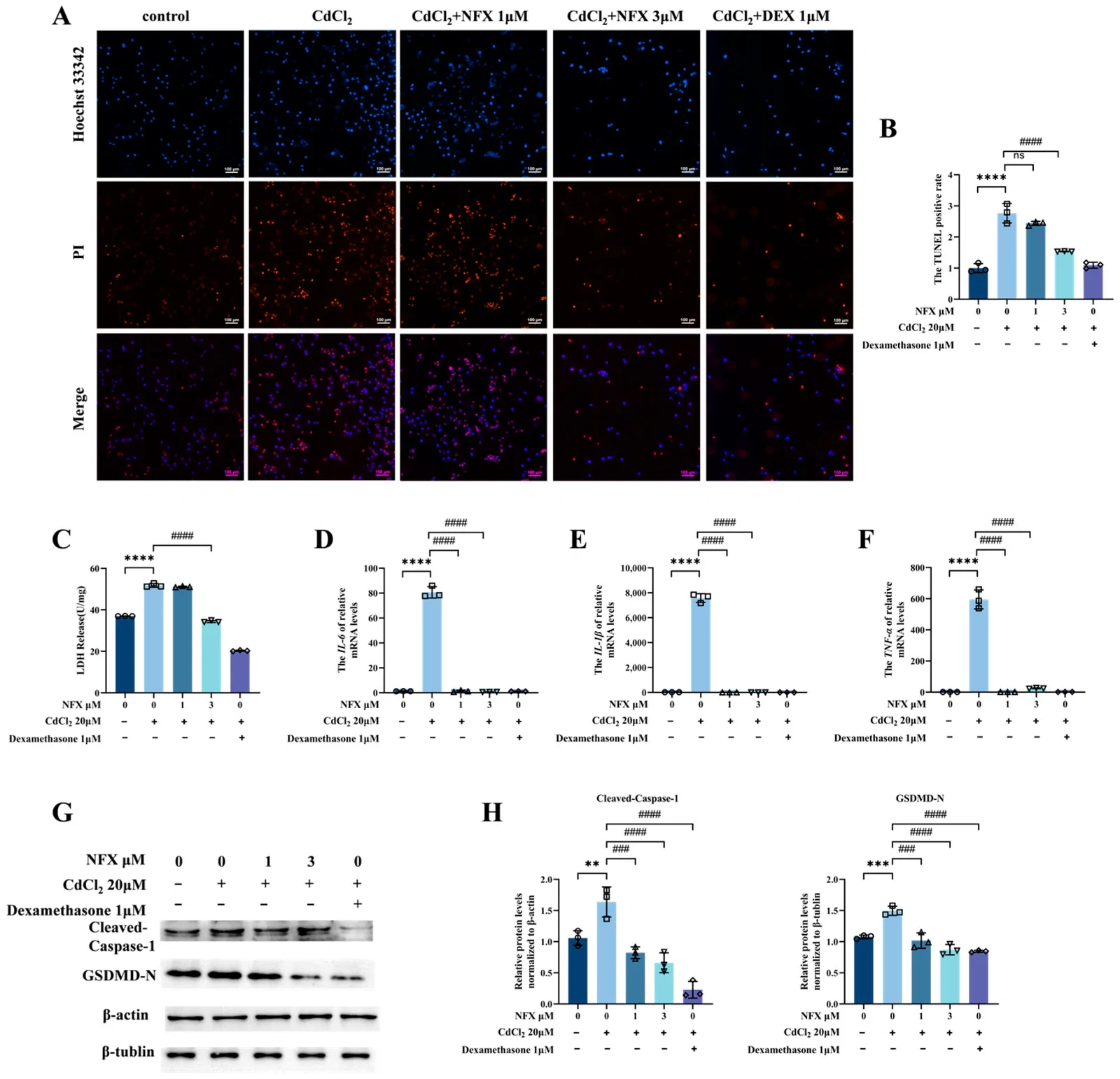
NFX attenuates CdCl_2_-induced hepatic stellate cell pyroptosis. (**A**,**B**) Fluorescence microscopy analysis of cell death. LX-2 cells were pretreated with serial concentrations of NFX (0, 1, 3 μM) for 2 h, followed by 20 μM CdCl_2_ stimulation for 24 h. Cells were co-stained with Hoechst 33342 (blue, nuclear staining) and propidium iodide (PI, red), and imaged under a fluorescence microscope. (**C**) Quantification of LDH release in cell culture supernatants. (**D**–**F**) qRT PCR detection of mRNA expression levels of pro inflammatory factors IL 6, IL 1β and TNF α. (**G**,**H**) Western blot analysis and densitometric quantification of cleaved caspase 1 and GSDMD N protein levels. LX 2 cells received 2 h pretreatment with NFX (0, 1, 3 μM) prior to 24 h exposure to 20 μM CdCl_2_, and whole cell lysates were subjected to immunoblotting. *n* = 3 independent experiments. Statistical analysis was performed using one-way ANOVA followed by Tukey’s post hoc test. ns, not significant vs. CdCl_2_ group; ^###^ *p* < 0.001, ^####^ *p* < 0.0001 vs. CdCl_2_ group; ** *p* < 0.01, *** *p* < 0.001, **** *p* < 0.0001 vs. control group.

To further validate the protective effects of NFX against CdCl_2_-induced liver injury and to elucidate its mechanism of action, we conducted in vitro experiments using human hepatic stellate cells (LX-2). Following a 2 h pretreatment with NFX at 0, 1, or 3 μM, cells were exposed to 20 μM CdCl_2_ for 24 h and then subjected to Hoechst 33342/propidium iodide (PI) double staining. Fluorescence microscopy revealed a pronounced increase in PI-positive cells (red fluorescence) after CdCl_2_ challenge, indicating increased cell death or late-stage apoptosis. In contrast, NFX pretreatment caused a dose-dependent decline in PI-positive cell counts (Figure 5A,B). LDH release assay and RT-qPCR further demonstrated that NFX dose-dependently reduced CdCl_2_-induced LDH release (Figure 5C) and downregulated intracellular mRNA levels of IL-6, IL-1β, and TNF-α in LX-2 cells (Figure 5D–F). Western blot analysis of pyroptosis hallmarks revealed that CdCl_2_ significantly increased the protein levels of cleaved caspase-1 and GSDMD-N; NFX treatment dose-dependently reduced the expression of both proteins (Figure 5G,H). These in vitro data collectively confirm that NFX effectively attenuates CdCl_2_-induced cell death in hepatic stellate cells.

### 3.5. USP21 Is a Candidate NFX-Interacting Protein and Functionally Implicated Target in Cadmium-Induced Hepatic Stellate Cell Injury

**Figure 6:**
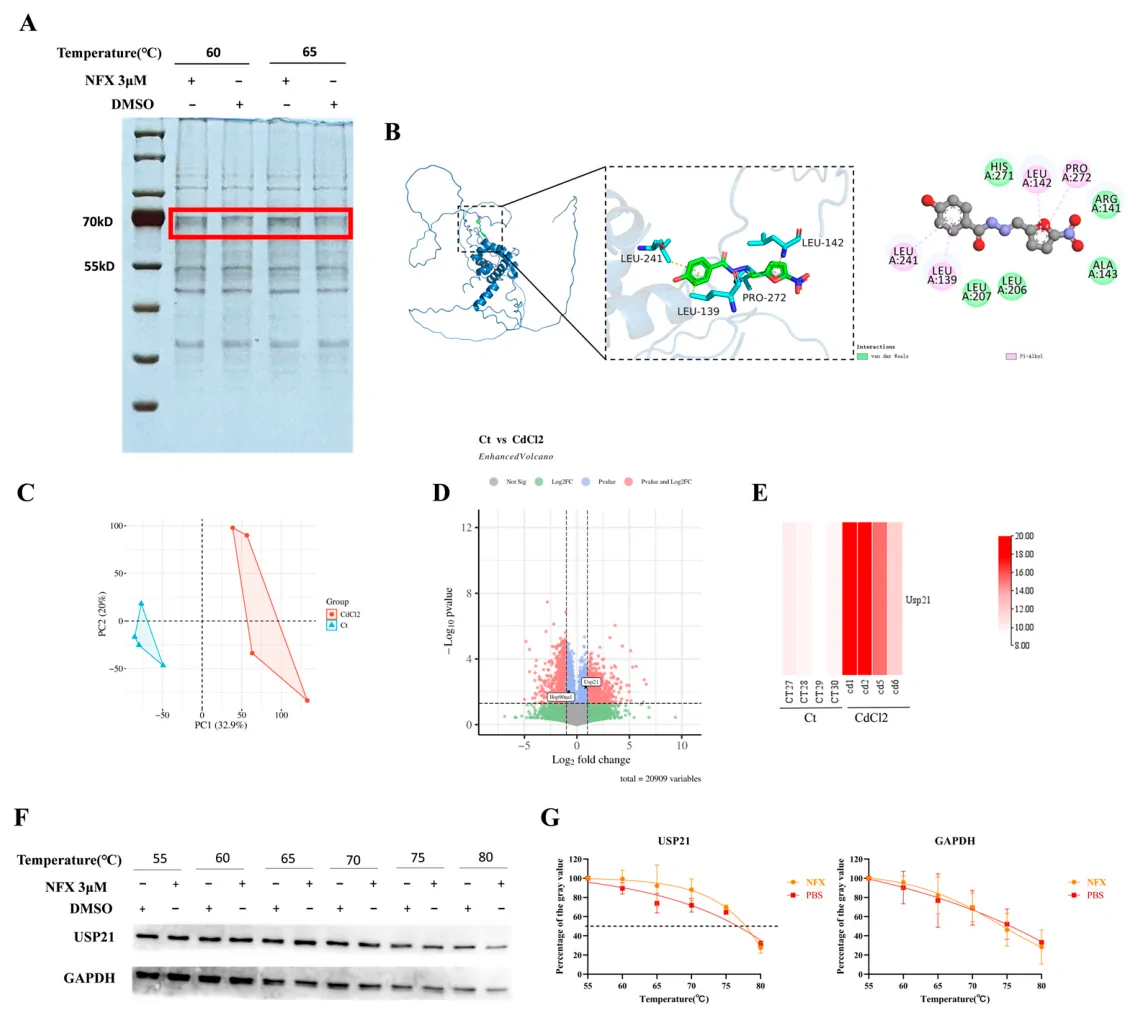
Identify the potential target of NFX. (**A**) Double temperature-point CETSA verifying the binding interaction between NFX and USP21. LX-2 cells were stimulated with 20 μM CdCl_2_ for 24 h, and the harvested cell lysates were equally divided into two aliquots. Lysates were incubated on ice for 30 min with either vehicle control (DMSO diluted in PBS) or 3 μM NFX solution, with identical final DMSO concentrations maintained in both groups. Protein thermal stability was detected at 60 °C and 65 °C. (**B**) Molecular docking model showing the potential binding interface and key interactive residues between NFX and USP21. (**C**) PCA analysis of partial GSE302882 dataset samples. (**D**) Volcano plot of differentially expressed genes after CdCl2 treatment. Volcano plot of differentially expressed genes after CdCl2 treatment. The vertical dashed lines indicate the thresholds of log2 fold change = −1 and 1, and the horizontal dashed line indicates the significance threshold of −log10(p value) = 1.301 (p = 0.05). (**E**) USP21 expression in samples. (**F**) Full-spectrum CETSA thermal stability curves of USP21. Cell lysates were treated with vehicle or 3 μM NFX as described above, and subjected to a temperature gradient ranging from 55 °C to 80 °C with an interval of 5 °C (six temperature points in total) to evaluate the thermal stabilization effect of NFX on USP21. (**G**) Densitometric quantification of relative USP21 protein levels normalized to GAPDH. *n* = 3 independent experiments.

To identify candidate molecular mediators of the hepatoprotective effect of NFX, we performed target screening and validation using the cellular thermal shift assay (CETSA). In the initial screening, silver staining revealed a candidate protein band at approximately 70 kDa (Figure 6A). Compared with the DMSO control group, this band exhibited enhanced thermal stability and reduced degradation in the presence of NFX, suggesting that it might represent an NFX-interacting protein candidate.

**Figure 7:**
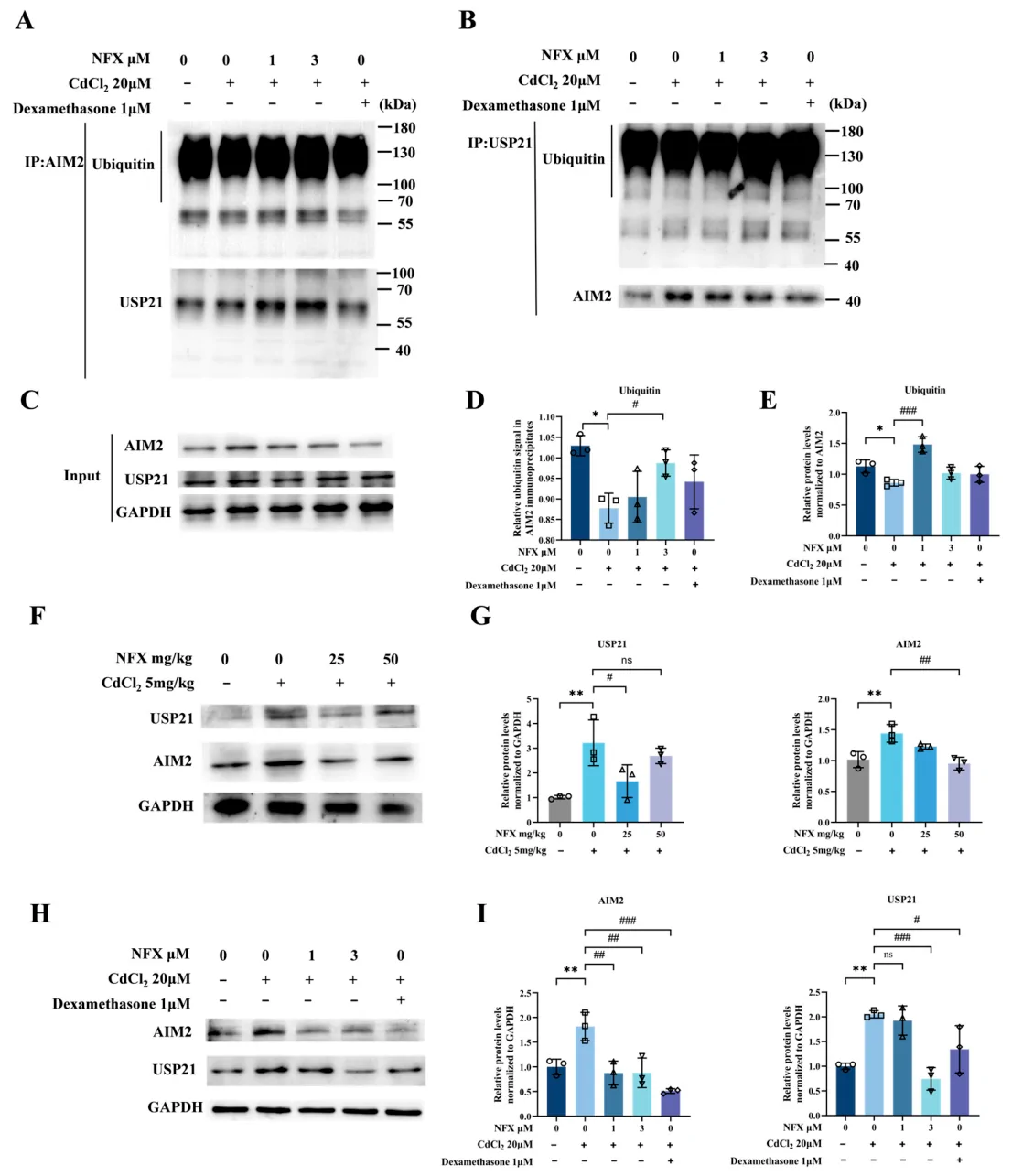
NFX alleviates CdCl_2_-induced pyroptosis by modulating the interaction between USP21 and AIM2. (**A**) Co-IP assay detecting the interaction between AIM2 and USP21. LX-2 cells were pretreated with NFX (0, 1, 3 μM) for 2 h, followed by 24 h of 20 μM CdCl_2_ stimulation and 6 h of 10 μM MG132 intervention before sample collection, *n* = 3 independent experiments. Cell lysates were immunoprecipitated with anti-AIM2 antibody, followed by immunoblotting with anti-USP21 and anti-ubiquitin antibodies. (**B**–**E**) Co-IP assay and corresponding quantitative analysis of AIM2 ubiquitination levels. LX-2 cells were treated as described above, *n* = 3 independent experiments. Cell lysates were immunoprecipitated with anti-USP21 antibody, and the precipitated complexes were immunoblotted using anti-AIM2 and anti-ubiquitin antibodies (**B**). Quantification of relative ubiquitin signal in anti-AIM2 immunoprecipitates. Equal volumes of immunoprecipitated eluates were loaded per lane, with all IP reactions initiated from equal amounts of total cell lysate protein (**D**). Densitometric quantification of AIM2 ubiquitination levels was performed (**E**). (**F**,**G**) Western blot analysis and densitometric quantification of USP21 and AIM2 protein expression in mouse liver tissues. Mice were intragastrically administered with NFX (25 mg/kg or 50 mg/kg) dissolved in 0.5% CMC-Na for three consecutive days. Two hours after the last NFX treatment, mice were intraperitoneally injected with 5 mg/kg CdCl_2_ diluted in normal saline. Liver tissues were collected 12 h after CdCl_2_ injection, immediately snap-frozen in liquid nitrogen and stored at −80 °C. Tissue homogenates were prepared, centrifuged, mixed with loading buffer, boiled, and subjected to immunoblotting, *n* = 3 mice per group. (**H**,**I**) Western blot analysis and densitometric quantification of USP21 and AIM2 protein levels in LX-2 cells. LX-2 cells were pretreated with gradient concentrations of NFX (0, 1, 3 μM) for 2 h and then exposed to 20 μM CdCl_2_ for 24 h before sample collection, *n* = 3 independent experiments. Statistical analysis was performed using one-way ANOVA followed by Tukey’s post hoc test. ns, not significant vs. CdCl_2_ group; ^#^ *p* < 0.05, ^##^ *p* < 0.01, ^###^ *p* < 0.001 vs. CdCl_2_ group; * *p* < 0.05, ** *p* < 0.01 vs. control group.

Previous studies have demonstrated that NFX directly binds to USP21 and inhibits its deubiquitinase activity in HepG2 cells [44], and USP21 plays important roles in pyroptosis, particularly in inflammasome regulation [24,45,46]. Further analysis of the publicly available transcriptomic dataset GSE302882 for acute cadmium exposure revealed elevated USP21 expression in the cadmium-exposed group (Figure 6C–E), which was consistent with our in vivo and in vitro observations that USP21 protein levels increased concomitantly with cadmium-induced liver injury and hepatic stellate cell activation (Figure 7F–I). Based on this expression correlation and known functional roles, we hypothesized that USP21 may be a functional mediator through which NFX exerts protective effects in this model.

Molecular docking analysis indicated that NFX forms hydrophobic interactions with key residues of USP21, including Leu139, Leu142, Leu241, and Pro272, which may serve as the main driving force for complex formation (Figure 6B). To further validate the interaction between NFX and USP21, we performed targeted CETSA experiments. The results showed that the thermal stability of USP21 was enhanced in the presence of NFX with increasing temperature, as reflected by a marked rightward shift in the melting curve (Figure 6F,G), which is consistent with a specific protein–ligand interaction. Collectively, these findings identify USP21 as a candidate NFX-interacting protein that is functionally implicated in cadmium-induced hepatic stellate cell injury.

### 3.6. NFX Modulates AIM2 Ubiquitination via USP21 in CdCl_2_-Induced Acute Liver Injury

In resting macrophages, AIM2 is known to undergo constitutive ubiquitination and proteasomal degradation, a mechanism that suppresses spontaneous inflammation. Upon DNA stimulation, however, USP21 associates with AIM2 and removes its ubiquitin chains, thereby elevating its protein stability [25]. In the preceding sections, NFX was found to alleviate CdCl_2_-induced pyroptosis. To explore the upstream mechanism underlying this effect, we selected USP21 as a candidate upstream regulator for further validation. We therefore examined whether the USP21–AIM2 axis operates in CdCl_2_-treated human hepatic stellate cell line LX-2. Co-IP experiments confirmed a physical interaction between USP21 and AIM2 (Figure 7A–C). To directly assess AIM2 ubiquitination, we performed AIM2-specific immunoprecipitation followed by immunoblotting with an anti-ubiquitin antibody (IP: AIM2; IB: ubiquitin). Total AIM2 ubiquitination was significantly reduced in the CdCl_2_ group compared with controls, whereas NFX co-treatment reversed this reduction and markedly increased AIM2 ubiquitination relative to the CdCl_2_ group (Figure 7A). In parallel, Western blot analysis showed that CdCl_2_ exposure significantly upregulated both USP21 and AIM2 protein levels in liver tissues and LX-2 cells, whereas NFX co-treatment significantly downregulated both USP21 and AIM2 protein levels. (Figure 7F–I). Collectively, these findings support a model in which CdCl_2_ upregulates USP21, leading to AIM2 deubiquitination and accumulation, whereas NFX counteracts this process by restoring AIM2 ubiquitination and reducing AIM2 protein levels.

### 3.7. USP21 Knockdown Enhances AIM2 Ubiquitination and Attenuates Cadmium-Induced Pyroptsis in LX-2 Cells

**Figure 8:**
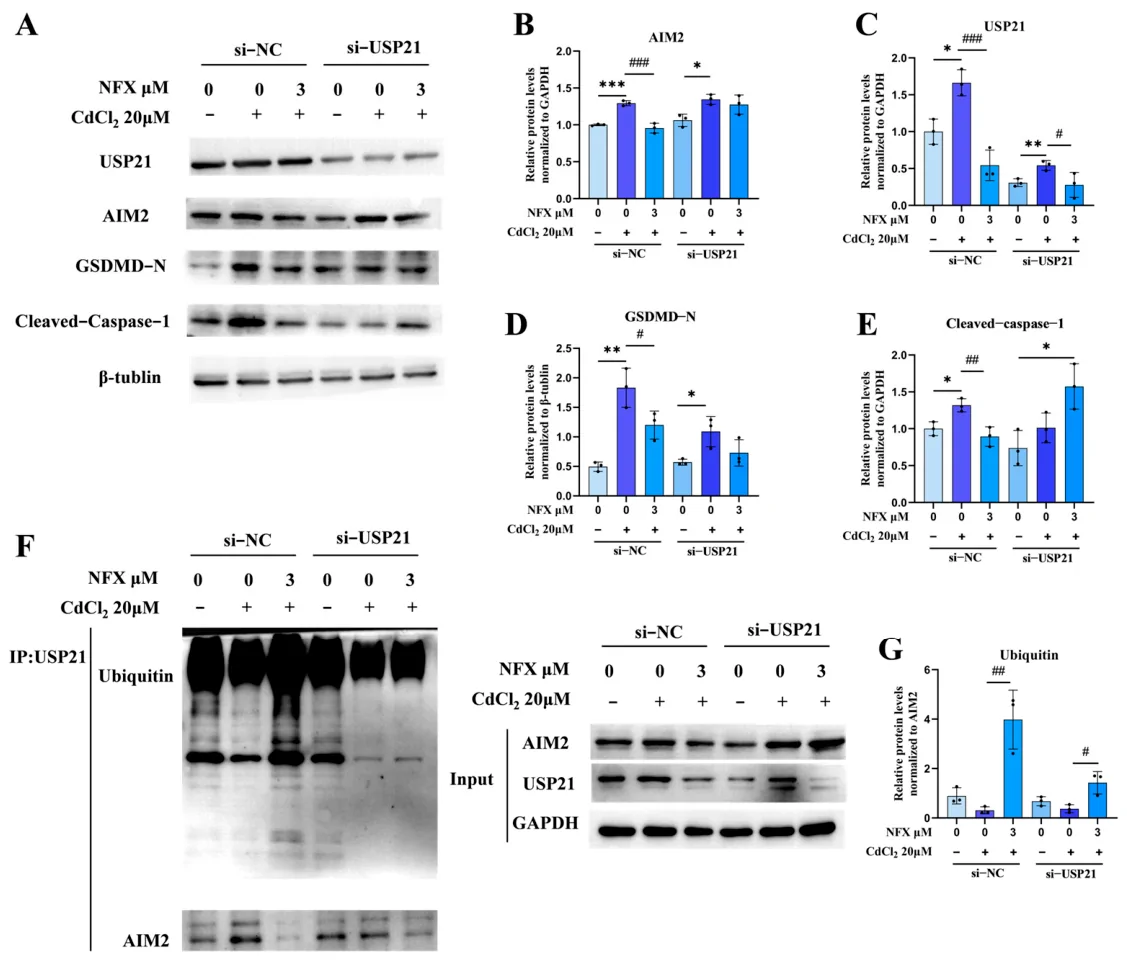
USP21 Knockdown Enhances AIM2 Ubiquitination and Attenuates Cadmium-Induced Pyroptosis in LX-2 Cells. (**A**) Western blot analysis of USP21, AIM2, GSDMD-N and cleaved-caspase-1 protein levels. LX-2 cells were seeded in 6-well plates and transfected with si-NC or si-USP21 when reaching 60–70% confluence. At 22 h post-transfection, cells were pretreated with 3 μM NFX for 2 h, followed by 20 μM CdCl_2_ stimulation for 24 h, and whole-cell lysates were harvested for immunoblotting. β-tubulin was used as the loading control for GSDMD-N and cleaved-caspase-1. (**B**–**E**) Densitometric quantification of relative protein abundance for AIM2 (**B**), USP21 (**C**), GSDMD-N (**D**), and cleaved-caspase-1 (**E**). Band intensities were quantified using ImageJ software. AIM2 and USP21 were normalized to GAPDH; GSDMD-N and cleaved-caspase-1 were normalized to β-tubulin. All data were further normalized to the si-NC control group. (**F**) Co-immunoprecipitation assay to detect USP21-AIM2 interaction and the ubiquitination status of USP21-associated AIM2. LX-2 cells were transfected as described above. At 22 h post-transfection, cells were pretreated with 3 μM NFX for 2 h and then exposed to 20 μM CdCl_2_ for 24 h. Cells were treated with 10 μM MG132 for 6 h prior to sample collection. Cell lysates were immunoprecipitated with anti-USP21 antibody, followed by immunoblotting for AIM2 and ubiquitin. (**G**) Quantitative analysis of ubiquitination levels of AIM2 recovered from USP21 immunoprecipitation. *n* = 3 independent experiments. Statistical significance markers shown in this figure are derived from one-way ANOVA followed by Tukey’s post hoc test across all six experimental groups. ^#^ *p* < 0.05, ^##^ *p* < 0.01, ^###^ *p* < 0.001 vs. CdCl_2_ group; * *p* < 0.05, ** *p* < 0.01, *** *p* < 0.001 vs. control group. Separate two-way ANOVA was performed exclusively on Cd-exposed subgroups to assess siRNA-by-NFX interaction; full main-effect and interaction *p*-values are provided in Table S3.

To investigate whether USP21 mediates the protective effect of NFX against cadmium-induced AIM2 inflammasome activation and pyroptosis, we knocked down USP21 in LX-2 cells via siRNA transfection and examined cellular responses to CdCl_2_ challenge with or without NFX pretreatment. Western blotting confirmed efficient knockdown of USP21 protein in si-USP21-transfected cells (Figure 8A,C). In si-NC control cells, CdCl_2_ exposure markedly upregulated the protein levels of AIM2, GSDMD-N, and cleaved caspase-1, while NFX treatment partially attenuated these increases (Figure 8A–E). In USP21-knockdown cells, CdCl_2_-induced upregulation of AIM2 and GSDMD-N was substantially blunted compared with the si-NC group, and the additional inhibitory effect of NFX was visibly reduced.

To formally test whether the regulatory effects of NFX depend on USP21 under cadmium challenge, we performed two-way ANOVA on the four Cd-exposed subgroups for each endpoint; full statistical outputs including main effects and interaction terms are summarized in Table S3. Significant siRNA-by-NFX interactions were observed for AIM2 protein abundance (*p* = 0.0053), USP21 expression (*p* = 0.0240), and ubiquitin signals in USP21 immunoprecipitates (*p* = 0.0388), indicating that NFX modulation of these targets is at least partially USP21-dependent. For GSDMD-N, both USP21 knockdown (*p* = 0.0063) and NFX treatment (*p* = 0.0359) exerted significant main effects, but the siRNA-by-NFX interaction did not reach statistical significance (*p* = 0.2247), suggesting that NFX regulates GSDMD-N partially through USP21-independent pathways. For cleaved caspase-1, the siRNA-by-NFX interaction was significant (*p* = 0.0204) despite non-significant main effects of siRNA (*p* = 0.5139) and NFX (*p* = 0.2314), consistent with a USP21-dependent regulatory pattern. Collectively, these statistical results support that NFX exerts its anti-inflammatory and anti-pyroptotic actions at least in part through a USP21-dependent mechanism.

To further examine the interplay between USP21 and the ubiquitination status of its associated protein complex, we performed co-immunoprecipitation with an anti-USP21 antibody and detected ubiquitin signals and co-precipitated AIM2 in the immunocomplexes (Figure 8F,G). In si-NC-transfected cells, CdCl_2_ stimulation significantly decreased the ubiquitin level in USP21 immunoprecipitates, while NFX co-treatment reversed this reduction. In USP21-knockdown cells, CdCl_2_ still reduced the ubiquitin signal, and NFX still promoted its recovery, but the magnitude of restoration was markedly attenuated compared with the si-NC group. In line with the two-way ANOVA interaction result for ubiquitin (*p* = 0.0388), these findings indicate that USP21 contributes to, but is not solely responsible for, NFX-mediated regulation of the ubiquitination status of the USP21-associated protein complex.

## 4. Discussion

Cadmium is a widespread environmental contaminant that accumulates primarily in the liver and kidneys. We acknowledge that in humans, the kidney is the principal site of chronic accumulation and long-term toxicity, whereas hepatotoxicity is less prominent under chronic exposure conditions. However, the present study employed acute CdCl_2_ challenge strictly as a mechanistic model to dissect the USP21–AIM2 inflammasome axis in hepatic stellate cell inflammatory signaling, rather than to recapitulate chronic cadmium toxicity in human populations.

This focus on the liver is supported by two key considerations. First, the liver is a primary target organ in acute cadmium poisoning and develops rapid, robust hepatocellular injury in animal models [3,4,5,6,47,48], providing a sensitive in vivo system for evaluating inflammasome-mediated pathology. Second, our core mechanistic investigation centers on LX-2 human hepatic stellate cells, which are predominantly liver-resident cells and represent a well-characterized cellular system for dissecting inflammasome-related signal transduction [30,49,50].

Using this approach, we investigated the protective mechanism of nifuroxazide (NFX) against cadmium-induced acute hepatotoxicity and identified a novel NFX–USP21–AIM2–pyroptosis axis underlying its prophylactic protective efficacy. This work also reveals a previously unrecognized role for USP21 as a positive regulator of AIM2 inflammasome protein stability. NFX pretreatment substantially ameliorated CdCl_2_-induced acute hepatic injury, including attenuation of lobular atrophy, inflammatory infiltration, and hepatocyte necrosis, accompanied by restoration of serum transaminase levels toward normal ranges. Mild histological improvements were also observed in the spleen and kidney, indicative of a systemic protective trend; however, these extrahepatic effects were moderate in magnitude and are not the primary focus of this mechanistic study.

Cadmium is known to disrupt hepatic redox balance [51,52,53,54,55]. In line with this, our results showed that CdCl_2_ treatment significantly reduced total antioxidant capacity (T-AOC), glutathione peroxidase (GSH-PX), and superoxide dismutase (SOD) activities, while elevating malondialdehyde (MDA) levels—indicative of severe oxidative damage. NFX administration effectively restored GSH-PX/SOD/T-AOC and curtailed MDA accumulation, suggesting that reinstatement of redox equilibrium constitutes a fundamental mechanism of NFX-mediated hepatoprotection. This antioxidant property is consistent with prior reports demonstrating the free radical–scavenging capacity of NFX [56,57].

The key innovation of this study lies in the identification of USP21 as a candidate functional target of NFX through the cellular thermal shift assay combined with molecular docking, in conjunction with functional validation. Strikingly, consistent with the canonical function of deubiquitinases that stabilize their substrates by removing ubiquitin chains [58,59,60,61], our data support that USP21 interacts with AIM2 and USP21 stabilizes AIM2 protein by removing its ubiquitin chains, likely including degradative chains, thereby increasing its intracellular abundance. As AIM2 is a critical sensor for pyroptosis, USP21-mediated stabilization of AIM2 ultimately promotes inflammasome activation and downstream pyroptotic signaling. Consistent with this functional role, our USP21 knockdown experiments showed that the ability of NFX to restore AIM2 ubiquitination and limit AIM2 accumulation was substantially attenuated when USP21 expression was reduced, confirming that USP21 is a major mediator of this regulatory axis. Although these findings establish a functional link between USP21 and AIM2, the precise molecular basis of USP21-mediated AIM2 regulation remains to be fully defined.

Previous research by our team has demonstrated that LECT2 levels are significantly elevated in infectious inflammation and exert a protective effect by activating macrophages [62,63]. In this study, elevated LECT2 levels were observed in the serum of mice in the CdCl_2_ model group, suggesting that LECT2 upregulation may represent a common stress response in various forms of acute liver injury, and providing new insights for future research on LECT2. In our model, CdCl_2_-driven USP21-mediated deubiquitination stabilizes AIM2 protein, expanding the pool of AIM2 available for assembly with ASC and caspase-1. Consistent with this model, NFX treatment profoundly suppressed CdCl_2_-induced caspase-1 activation, GSDMD-N generation, and release of IL-1β and IL-18—all hallmarks of pyroptosis [64,65,66]. Importantly, unlike conventional small-molecule caspase-1 inhibitors that act on terminal effectors, NFX acts upstream by reducing AIM2 protein abundance and limiting inflammasome activation, offering a conceptually novel anti-inflammatory strategy [51,67,68].

Co-immunoprecipitation revealed a physical USP21–AIM2 interaction and showed that ubiquitination of USP21-bound AIM2 is dynamically regulated by cadmium exposure and NFX treatment. In control si-NC cells, cadmium markedly reduced AIM2 ubiquitination within the USP21-associated complex, accompanied by increased AIM2 abundance and inflammasome activation. NFX co-treatment counteracted this cadmium-induced deubiquitination, restoring AIM2 ubiquitination and suppressing AIM2 accumulation and pyroptotic signaling.

These data support a model in which USP21, a deubiquitinating cysteine protease, may directly modulate AIM2 ubiquitination and stability. Cadmium may enhance USP21 deubiquitinase activity toward AIM2, thereby reducing AIM2 ubiquitination and proteasomal degradation and increasing its steady-state levels. NFX may protect, at least in part, by inhibiting USP21 deubiquitinase activity, maintaining AIM2 ubiquitination and limiting its excessive accumulation and subsequent inflammasome activation. USP21 knockdown substantially attenuated NFX-mediated restoration of AIM2 ubiquitination. A similar trend persisted in USP21-depleted cells, but the magnitude of recovery was clearly weaker than in controls, indicating that USP21 is a major but not exclusive mediator. Other deubiquitinases or regulatory pathways likely contribute, consistent with the partial rather than complete attenuation of NFX’s protective effects on pyroptosis.

**Figure 9:**
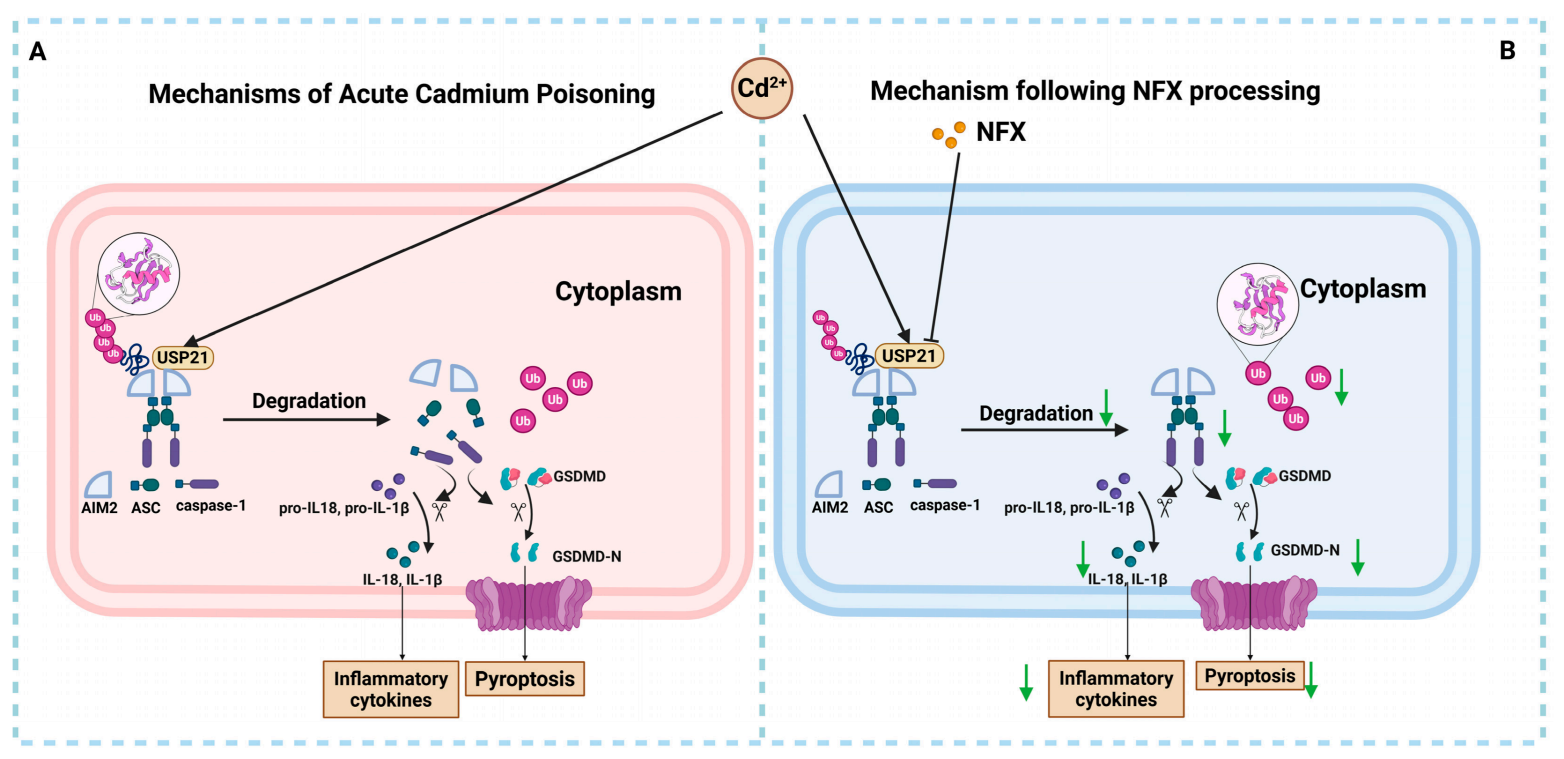
Schematic diagram illustrating how NFX alleviates CdCl_2_-induced acute liver injury in mice via the USP21–AIM2 pyroptosis axis in hepatic stellate cells. (**A**) Mechanisms of acute cadmium poisoning. (**B**) Mechanism following NFX processing. Black arrows indicate activation, promotion, or transformation; green downward arrows indicate attenuation of the process or reduced generation of certain small molecules; the arrow labeled “Degradation” indicates protein degradation. The pink and blue backgrounds are used only to distinguish the two treatment conditions, and the orange dots represent NFX. Created in BioRender. Yang, G. (2026) https://BioRender.com/qkopryu, accessed on 28 July 2026.

Collectively, this study confirms that NFX pretreatment alleviates CdCl_2_-induced acute liver injury via modulation of the USP21/AIM2 signalling pathway (Figure 9). These findings suggest that NFX, as a molecule with organ-protective effects, may offer a potential strategy for prophylactic protection against acute cadmium exposure. Furthermore, we found that USP21 is a key regulator of AIM2 inflammasome stability, acting as a positive modulator by deubiquitinating and stabilizing AIM2 protein; this provides new insights for the development of anti-inflammatory and organ-protective intervention strategies.

## 5. Limitations

Notwithstanding the protective effects of NFX against cadmium-induced hepatic injury and the identification of the USP21/AIM2 regulatory axis presented herein, several limitations of the study warrant acknowledgment. First, while our data demonstrate that USP21 interacts with AIM2 and stabilizes the protein by removing its ubiquitin chains, the precise molecular details of this process remain incompletely characterized. The ubiquitination signal measured via anti-USP21 immunoprecipitation reflects only the USP21-bound AIM2 pool rather than total cellular AIM2, and thus primarily captures regulation at the USP21–AIM2 interface. For the anti-AIM2 immunoprecipitation assay used to directly assess AIM2 ubiquitination, AIM2 protein in the immunoprecipitated eluates was not re-probed for normalization, so we cannot fully exclude potential group-to-group variation in AIM2 pull-down efficiency. In addition, only total ubiquitination of AIM2 was detected rather than K48-linked ubiquitin chains, which are the canonical signal for proteasomal degradation, and AIM2 protein half-life experiments were not performed to directly confirm proteasome-dependent AIM2 degradation. The exact mechanism through which USP21 mediates AIM2 stability and turnover therefore requires further in-depth investigation. Second, NFX was administered in a prophylactic pretreatment regimen prior to cadmium challenge, rather than as a therapeutic intervention after exposure, and only an acute cadmium exposure model was evaluated. These study design choices constrain the direct translational relevance of our findings to real-world environmental or clinical settings of cadmium exposure, where intervention typically occurs after exposure has already taken place. Third, formal a priori power calculations were not performed for the in vivo experiments. Fourth, the CETSA-based target identification revealed a candidate band at approximately 70 kDa; although USP21 was prioritized on the basis of prior evidence and subsequent validation, it remains possible that other proteins of similar molecular weight also participate in the regulation of pyroptosis during acute cadmium exposure. Further studies are warranted to exclude or characterize the potential contribution of additional interacting proteins to the observed hepatoprotective effects.

Additionally, although TUNEL staining of liver tissue suggested a potential effect of NFX on hepatocyte apoptosis, our in vitro mechanistic experiments focused exclusively on LX-2 human hepatic stellate cells, aligned with the study’s core emphasis on inflammatory and pyroptotic signaling in non-parenchymal cells. Follow-up investigations using primary hepatocytes or hepatocyte cell lines will be needed to validate the hepatocellular effects of NFX and generalize the mechanism across hepatic cell types. With respect to data sources, analyses derived from public databases were used solely as supporting evidence for the observed phenotypes; the core mechanistic conclusions of this work are based entirely on experimentally validated findings regarding the USP21/AIM2 axis.

Overall, while these limitations constrain the mechanistic depth and translational scope of the current study, they do not invalidate the core finding that NFX ameliorates cadmium-induced acute liver injury at least in part by modulating the USP21/AIM2-pyroptosis axis. These unresolved issues also delineate clear directions for future research to further characterize the molecular mechanisms and translational potential of this regulatory pathway.

## 6. Conclusions

In summary, this study demonstrates that NFX pretreatment attenuates CdCl_2_-induced liver injury, at least in part via modulation of the USP21/AIM2 signaling pathway. These findings suggest that NFX may represent a potential preventive strategy against acute cadmium exposure. Furthermore, we identify USP21 as a novel positive regulator of AIM2 protein stability; USP21 stabilizes AIM2 by removing its ubiquitin chains to facilitate AIM2 inflammasome activation, which provides new insights for developing anti-inflammatory and organ-protective intervention strategies.

## Figures and Tables

**Table 1:** Sequences of si-USP21 used in this study.

siRNA	Sense Sequence (5′-3′)	Antisense Sequence (5′-3′)
si-USP21	GCAAGAUUGUGGACCUGUUTT	AACAGGUCCACAAUCUUGCTT

## Data Availability

The original contributions presented in this study are included in the article/Supplementary Material. Further inquiries can be directed to the corresponding authors.

## Supplementary Materials

The following supporting information can be downloaded at: https://www.mdpi.com/article/10.3390/antiox15091211/s1, Table S1: The primers used in this manuscript. All RT-qPCR primer sequences are listed. Table S2: The information about the antibodies used in this manuscript. Antibodies used for immunoblotting are listed. Table S3: Summary of two-way ANOVA results for Cd-challenged subgroups. Full statistical outputs of two-way ANOVA (main effects and interaction p-values) for the USP21 knockdown assay are summarized. Figure S1: Histopathological evaluation of spleen and kidney tissues in response to different treatments. Hematoxylin and eosin (H&E) staining results of kidney and spleen are shown. Uncropped Western blot images for all key proteins are presented in the Supplementary Data. Each full-length membrane image includes molecular weight markers, and the quantified regions are demarcated by red boxes.

## Abbreviations

The following abbreviations are used in this manuscript:

NFX	Nifuroxazide
SOD	Superoxide Dismutase
GSH-PX	Glutathione Peroxidase
MDA	Malondialdehyde
T-AOC	Total Antioxidant Capacity
AST	Aspartate Aminotransferase
ALT	Alanine Aminotransferase
α-SMA	Alpha-Smooth Muscle Actin
COL-1	Collagen Type I
COL-3	Collagen Type III
IL-1β	Interleukin-1β
TNF-α	Tumor Necrosis Factor-alpha
IL-18	Interleukin-18
ASC	Apoptosis-speck-like protein containing a CARD
IL-6	Interleukin-6
LDH	Lactate Dehydrogenase
GSDMD-N	Gasdermin D N-terminal fragment
USP21	Ubiquitin-Specific Peptidase 21
AIM2	Absent in Melanoma 2
ROS	Reactive Oxygen Species
TUNEL	TdT-UTP Nick End Labeling
DMSO	Dimethyl Sulfoxide
SDS-PAGE	Sodium Dodecyl Sulfate Polyacrylamide Gel Electrophoresis
RIPA	RadioImmunoprecipitation Assay buffer
BCA	Bicinchoninic Acid Assay
PVDF	Polyvinylidene Difluoride
