# Supplementary material for: Nifuroxazide Pretreatment Protects Against Acute CdCl2 Exposure-Induced Toxicity via Modulating the USP21/AIM2 Inflammasome Axis
Source: Antioxidants (Basel). 2026 Sep 20;15(9):1211. doi: 10.3390/antiox15091211 (PMC13603489; doi:10.3390/antiox15091211)
Supplement: Supplementary file 1 [file antioxidants-15-01211-s001.zip › antioxidants-4496676-supplementary.pdf]

Table S1. The primers used in this manuscript.

Primer	Gene	Sequence	Bp
Mouse-*IL-1β* (F)	*IL-1β*	CACTACAGGCTCCGAGATGAACAAC	25
Mouse-*IL-1β*(R)		TGTCGTTGCTTGGTTCTCCTTGTAC	24
Mouse-*TNF-α* (F)	*TNF α*	GGACTAGCCAGGAGGGAGAACAG	23
Mouse-*TNF-α* (R)		GCCAGTGAGTGAAAGGGACAGAAC	24
Mouse-*IL-6* (F)	*IL-6*	AGCCCACCAAGAACGATAGTCAATTC	26
Mouse-*IL-6* (R)		GTCACCAGCATCAGTCCCAAGAAG	24
Mouse-*IL-18* (F)	*IL-18*	GACTCTTGCGTCAACTTCAAGG	22
Mouse-*IL-18* (R)		CAGGCTGTCTTTTGTCAACGA	21
Mouse-*ASC* (F)	*ASC*	CTTGTCAGGGGATGAACTCAAAA	23
Mouse-*ASC* (R)		GCCATACGACTCCAGATAGTAGC	23
Mouse-*Caspase-1* (F)	*Caspase-1*	ACAAGGCACGGGACCTATG	19
Mouse-*Caspase-1* (R)		TCCCAGTCAGTCCTGGAAATG	21
Human-*IL-1β* (F)	*IL-1β*	CAACCAACAAGTGATATTCTCCATG	25
Human-*IL-1β* (R)		GATCCACACTCTCCAGCTGCA	21
Human-*IL-6* (F)	*IL-6*	CGGGAACGAAAGAGAAGCTCTA	22
Human-*IL-6* (R)		GAGCAGCCCCAGGGAGAA	18
Human-*TNF-α*(F)	*TNF-α*	CATCTTCTCAAAATTCGAGTGACAA	25
Human-*TNF-α*(R)		TGGGAGTAGACAAGGTACAACCC	23
Human-*β-actin* (F)	*β-actin*	CGCCGCCAGCTCACCATG	18
Human-*β-actin* (R)		CACGATGGAGGGGAAGAC	18
Mouse-*β-actin* (F)	*β-actin*	ATGGATGACGATATCGCTG	19
Mouse-*β-actin* (R)		GTTGGTAACAATGCCATGTTC	21

Table S2. The information about the antibodies used in this manuscript.

Antibody name	Species	Dilution ratio	Catalogue No.	Brand
AIM2	Rabbit	1:1000	CSB-PA001499LA01HU	CUSABIO
USP21	Rabbit	1:1000	A16663	Abclonal
Caspase1	Rabbit	1:1000	HA722222	HUABIO
GSDMD	Rabbit	1:1000	HA721144	HUABIO
Ubiquitin	Rabbit	1:1000	AB2041	Beyotime
GADPH	Mouse	1:3000	AC035	ABclonal
β-Tubulin	Mouse	1:3000	AC021	ABclonal
Anti-Rabbit IgG	Goat	1:5000	BF03008	Biodragon
Anti-Mouse IgG	Goat	1:5000	BF03001	Biodragon

Table S3. Summary of two-way ANOVA results for Cd-challenged subgroups

Protein	Main effect of siRNA (P-value)	Main effect of NFX (P-value)	siRNA-by-NFX Interaction (P-value)
AIM2	0.0011	0.0414	0.0053
USP21	0.0003	0.0046	0.0240
Cleaved-Caspase1	0.5139	0.2314	0.0204
GSDMD-N	0.0063	0.0359	0.2247
Ubiquitin	0.0015	0.0451	0.0388

Table S3 Summary of two-way ANOVA outputs performed on four Cd-exposed experimental subgroups (si-NC+$CdCl_2$, si-NC+ $CdCl_2$+NFX, si-USP21+ $CdCl_2$, si-USP21+ $CdCl_2$+NFX). Two-way ANOVA was used to test main effects of siRNA status (si-NC vs si-USP21), main effects of NFX treatment (− NFX vs + NFX), and siRNA-by-NFX interaction term. Untreated blank control groups without Cd exposure were excluded from this analysis.

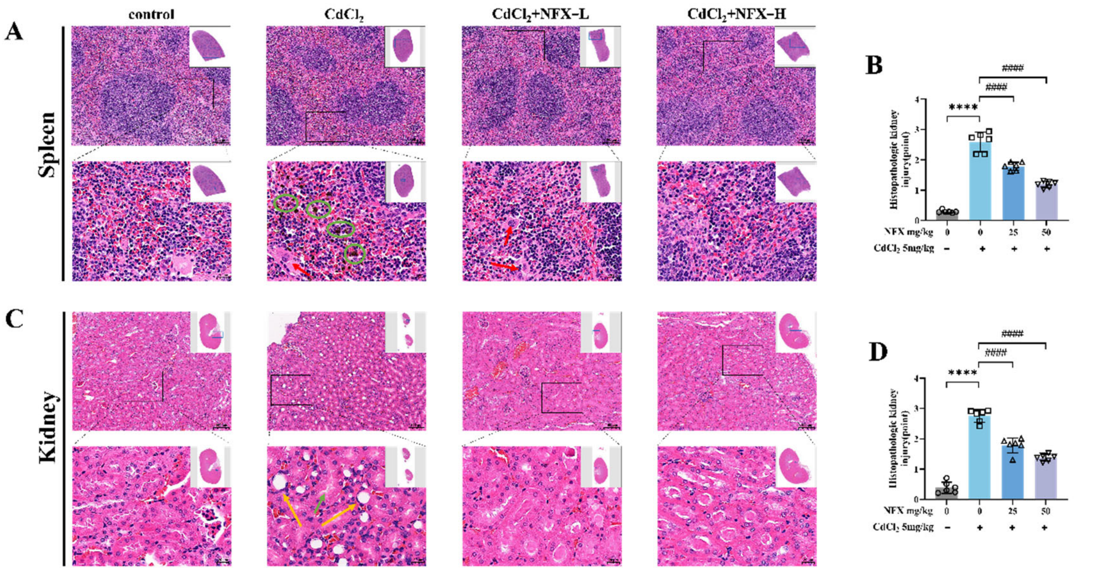

Figure S1. Histopathological evaluation of spleen and kidney tissues in response to different treatments. (A) Histopathological sections of mouse spleen under different treatment conditions (H&E staining). For each organ, the top image in each set shows the field of view under low magnification (10×), whilst the bottom image shows a high-magnification view (40×) of the boxed area; (B) Histopathological scoring of H&E-stained spleen sections; (C) Histopathological sections of mouse kidney under different treatment conditions (H&E staining). For each organ, the top image in each set shows the field of view under low magnification (10×), whilst the bottom image shows a high-magnification view (40×) of the boxed area; (D) Histopathological scoring of H&E-stained kidney sections. Spleen section: ○ highlights areas of haem deposition, → indicates extramedullary haematopoietic foci; Kidney section: → indicates areas of lipid degeneration, → highlights areas of amyloid deposits. The six data points shown in the figure represent 3 animals × 2 fields per animal; statistical analysis was based on the average of the two fields from each animal, with n = 3 animals per group. $^{\#}p < 0.05$, $^{\#\#}p < 0.01$, $^{\#\#\#}p < 0.001$, $^{\#\#\#\#}p < 0.0001$ vs $CdCl_2$ group; $^{*}p < 0.05$, $^{**}p < 0.01$, $^{***}p < 0.001$, $^{****}p < 0.0001$ vs control group.
